# Letter from the Editor in Chief

**DOI:** 10.19102/icrm.2023.14046

**Published:** 2023-04-15

**Authors:** Moussa Mansour


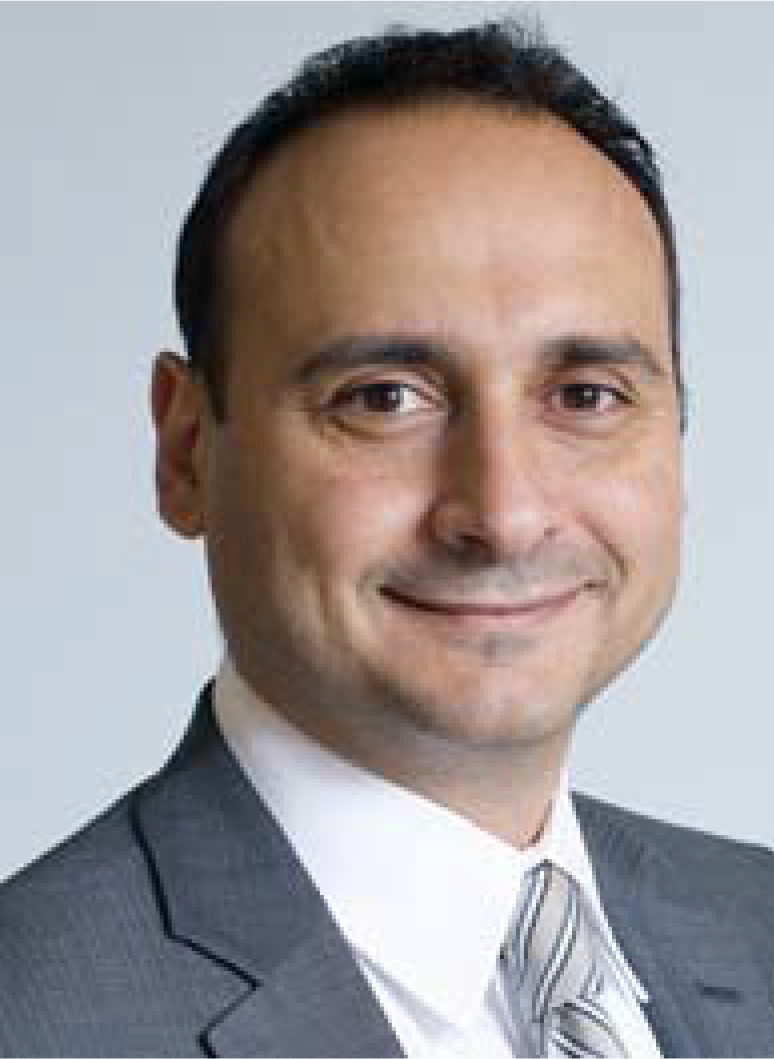


Dear readers,

Vascular access complications during electrophysiology procedures are some of the most common adverse events. They can range from hematomas causing pain, discomfort, and increased hospital stay lengths to retroperitoneal bleeding and hemothorax that may lead to death. Over the past decade, various techniques have been used to reduce the rate of vascular access complications, including the use of micro-puncture needles, closure devices, and—most importantly—ultrasound guidance.

In this issue of *The Journal of Innovations in Cardiac Rhythm Management*, there is an important article by Maffè et al. titled “Ultrasound-guided Axillary Vein Puncture for Cardiac Device Implantation: A Safe and Effective Approach.”^1^ In it, the authors report the safety, efficacy, and radiation exposure of an ultrasound-guided axillary approach compared to other conventional access techniques. They analyzed the outcomes of 65 patients who underwent ultrasound-guided axillary vein access and compared them to those of 65 control patients. The ultrasound-guided approach was found to be associated with reduced radiation exposure and shorter procedure times. Complications occurred in 2 study group patients (2 with axillary artery puncture) and 6 control group patients (1 with an urticaria contrast medium–related event, 3 with pneumothorax, and 2 with subclavian artery puncture).

The above-mentioned study has some limitations due to it being a small, observational, and retrospective investigation. It remains an important piece of work, however, because it provides another piece of evidence in support of the use of ultrasound to guide the implantation of cardiovascular implantable electronic devices. A few other studies have reported similar findings, and most have demonstrated that ultrasound guidance leads to reduced procedure times and radiation exposure in addition to increased success in obtaining venous access, especially in challenging situations. In the current study and others, there has been a trend toward reduced complications associated with ultrasound guidance; however, demonstrating a statistically difference in the rate of complications would require a much larger study because of small event rates.

Ultrasound-guided femoral access has become the standard approach for ablation procedures in most centers in the United States. The data in support of using ultrasound guidance for axillary access are growing, and I believe this technique should become the standard as well.

I hope that you enjoy reading this issue of *The Journal of Innovations in Cardiac Rhythm Management*.


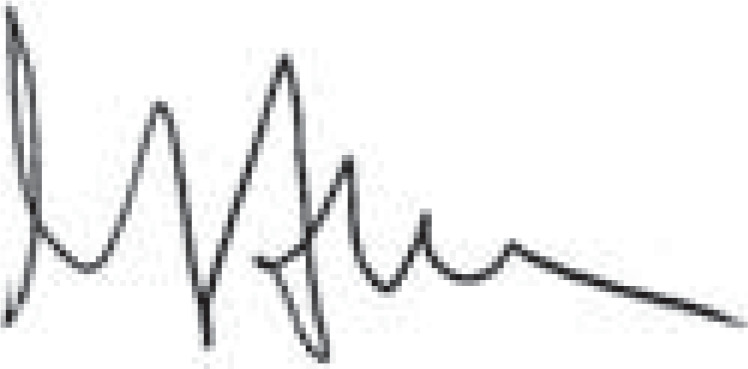


Sincerely,

Moussa Mansour, md, fhrs, facc

Editor in Chief

*The Journal of Innovations in Cardiac Rhythm Management*

MMansour@InnovationsInCRM.com

Director, Atrial Fibrillation Program

Jeremy Ruskin and Dan Starks Endowed Chair in Cardiology

Massachusetts General Hospital

Boston, MA 02114
